# Cardiac-Specific Expression of the Tetracycline Transactivator Confers Increased Heart Function and Survival Following Ischemia Reperfusion Injury

**DOI:** 10.1371/journal.pone.0030129

**Published:** 2012-01-17

**Authors:** Laila Elsherif, Xuerong Wang, Milana Grachoff, Beata M. Wolska, David L. Geenen, John P. O'Bryan

**Affiliations:** 1 Department of Pharmacology, College of Medicine, University of Illinois-Chicago, Chicago, Illinois, United States of America; 2 Section of Cardiology, Department of Medicine, College of Medicine, University of Illinois-Chicago, Chicago, Illinois, United States of America; 3 Department of Physiology and Biophysics, College of Medicine, University of Illinois-Chicago, Chicago, Illinois, United States of America; 4 Center for Cardiovascular Research, College of Medicine, University of Illinois-Chicago, Chicago, Illinois, United States of America; Istituto Dermopatico dell'Immacolata-IRCCS, Italy

## Abstract

Mice expressing the tetracycline transactivator (tTA) transcription factor driven by the rat α-myosin heavy chain promoter (α-MHC-tTA) are widely used to dissect the molecular mechanisms involved in cardiac development and disease. However, these α-MHC-tTA mice exhibit a gain-of-function phenotype consisting of robust protection against ischemia/reperfusion injury in both *in vitro* and *in vivo* models in the absence of associated cardiac hypertrophy or remodeling. Cardiac function, as assessed by echocardiography, did not differ between α-MHC-tTA and control animals, and there were no noticeable differences observed between the two groups in HW/TL ratio or LV end-diastolic and end-systolic dimensions. Protection against ischemia/reperfusion injury was assessed using isolated perfused hearts where α-MHC-tTA mice had robust protection against ischemia/reperfusion injury which was not blocked by pharmacological inhibition of PI3Ks with LY294002. Furthermore, α-MHC-tTA mice subjected to coronary artery ligation exhibited significantly reduced infarct size compared to control animals. Our findings reveal that α-MHC-tTA transgenic mice exhibit a gain-of-function phenotype consisting of robust protection against ischemia/reperfusion injury similar to cardiac pre- and post-conditioning effects. However, in contrast to classical pre- and post-conditioning, the α-MHC-tTA phenotype is not inhibited by the classic preconditioning inhibitor LY294002 suggesting involvement of a non-PI3K-AKT signaling pathway in this phenotype. Thus, further study of the α-MHC-tTA model may reveal novel molecular targets for therapeutic intervention during ischemic injury.

## Introduction

The tetracycline-controlled transactivator (tTA) expression system has been a widely used system for generating tissue-specific and temporally-regulated expression of gene targets in mouse models. The tTA-regulated system consists of two arms: one with tissue specific expression of the tTA transactivator and the other where the gene of interest is expressed under the control of a tTA responsive promoter element [1]. The cardiac specific tetracycline-regulated system consists of two transgenic lines: one expressing the tTA transactivator under the control of the rat α-MHC promoter and a second line possessing a target gene whose expression is controlled by a tTA-responsive promoter [2]. A double transgenic line created by crossing the two lines expresses the target gene in a cardiac-specific manner in the absence of tetracycline (Tet) or its analogue, doxycycline (Dox). In the presence of tetracycline, tTA and tetracycline form a complex that is unable to bind to the tTA responsive element leading to inhibition of transgene expression. This system allows for analysis of gene expression in adult animals without the confounding effects due to transgene expression during development.

Expression of tTA under the control of the rat α-MHC promoter (α-MHC-tTA) has allowed for the study of temporal and spatial expression of various important molecules in cardiac tissue [2], [3]. The important roles of key elements such FrzA/sFRP-1, PKC, nNOS and the glucocorticoid receptor in cardiac development and disease processes have been elucidated using the tTA-regulated expression system [2], [4], [5], [6], [7]. However, it has been shown recently that expression of the tTA transcription factor leads to a cardiomyopathy characterized by hypertrophy, ventricular dilation and decreased ejection fraction *in vivo*; whereas *in vitro* an increase in myofilament Ca^+^ sensitivity and in submaximal contraction were observed [8]. Furthermore, a sustained protective effect was observed in the α-MHC-tTA mice when subjected to ischemia/reperfusion (I/R) injury *in vitro* using the isolated mouse heart preparation [9]. This sustained effect was not abolished by inhibitors of mitochondrial ATP-sensitive K^+^ channel, PKC, or adenosine receptors. Although the preconditioning effect is a very interesting finding in the field of cardiac I/R injury, it is tempered by the concurrent impairment in cardiac function, and the presence of cardiac hypertrophy and ventricular dilation *in vivo*.

We report here for the first time a sustained protective effect in the α-MHC-tTA mice in the absence of hypertrophy, dilation, or cardiac dysfunction *in vivo*. We observed that α-MHC-tTA hearts had dramatically improved recovery of function compared to control hearts following I/R injury. The observed protective effect was not blocked by the pharmacological inhibitor LY294002 indicating that the protection was independent of the PI3K-AKT signaling pathway. Furthermore, I/R injury induced *in vivo* by coronary artery occlusion resulted in smaller infarcts in α-MHC-tTA compared to control indicating that the α-MHC-tTA mice are also protected from cell death *in vivo*. The present model offers a promising finding in the field of cardiac I/R injury and for the development of novel therapeutic agents especially with the advancement of the technology of biologics in the pharmaceutical industry. Dissecting the mechanisms involved in this protective effect will greatly enhance our knowledge base of a very prevalent condition in the Western population.

## Materials and Methods

### Animals and ethics statement

All methods involving animal use conform with the *Guide for the Care and Use of Laboratory Animals* published by the National Institutes of Health (NIH publication no. 85-23, revised 1996) and were approved by the Animal Care and Use Committee of the University of Illinois at Chicago. Mice expressing the tetracycline-controlled transactivator (tTA) under the control of the rat α-myosin heavy chain (αMHC-tTA) were purchased from Jackson laboratories (stock number 003170). These mice, which were initially maintained on an FVB/N background, were backcrossed to C57BL/6 strain for multiple generations (at least 6). Transgenic mice were identified by PCR screening. The tTA protein is constitutively expressed in the myocardium and all mice were maintained on a tetracycline-free drinking water. Control mice were either non-transgenic C57BL/6 mice or mice possessing a tet-regulated intersectin transgene [10]. These tet-transgenic mice were maintained on a pure C57BL/6 background, do not express intersectin in the absence of tTA, and appear indistinguishable from pure-bred C57BL/6 mice. As with the tet-transgenic mice, the α-MHC-tTA-transgenic mice do not show any overt phenotype and appear indistinguishable from pure-bred C57BL/6 mice.

### Echocardiography

Transgenic male mice (5–9 months of age, 6 control and 5 αMHC-tTA-transgenic) were examined by echocardiography for baseline measurements of cardiac function. Mice were initially anesthetized with 3% isoflurane (Isoflurane, USP, Halocarbon Products Corporation) in oxygen in an induction chamber and maintained on isoflurane (1.0–1.5%) in oxygen using a nose cone [11]. They were placed in the decubitus position on a warming pad to maintain normothermia. The chest was shaved and hair was removed with a depilatory cream (Nair). Warmed (37°C) ultrasound gel (Aquasonic 100, Parker Laboratories, INC., Fairfield, N.J.) was applied to the chest. Transthoracic 2-D, M-mode and pulsed Doppler images were acquired using a high-resolution echocardiographic system (Vevo 770, Visual Sonics, Toronto, ON, Canada) equipped with a 30-MHz mechanical transducer. Left ventricular (LV) end-diastolic (LVIDd) and end-systolic (LVIDs) diameters were obtained from the M-mode in parasternal short axis view of LV at mid-papillary level, and fractional shortening was calculated as FS% = (LVIDd-LVIDs)/LVIDd×100%. All measurements were according to the leading-edge method of the American Society of Echocardiography and were based on the average of at least three cycles. LV end-diastolic (EDV) and end-systolic (ESV) volumes were calculated from the parasternal long axis and short axis views using the area-length method as following: EDV = 5/6×Ad×Ld, where Ad, Ld are area and length, respectively, of the LV in end-diastole; ESV = 5/6×As×Ls, where As, Ls are area and length of the LV in end-systole. Ejection fraction was calculated as EF% = (EDV-ESV)/EDV.

### Langendorff-perfused heart and ischemia/reperfusion injury experimental protocol

For Langendorff-perfused heart experiments, 3 control and 4 αMHC-tTA mice age 3–5 months were used. Mice were injected with 200 U (IP) of heparin 20 minutes prior to IP administration of sodium pentobarbital (70 mg/kg). The heart was rapidly excised and placed on ice-cold modified Krebs-Henseleit (KH) buffer containing (in mM): 118.5 NaCl, 25 NaHCO_3_, 4.7 KCl, 1.2 MgSO_4_, 1.2 KH_2_PO_4_, 11 glucose, 2.5 CaCl_2_, 0.5 EDTA, 2 Na Pyruvate; buffer was bubbled with 5% CO_2_/95% O_2_ at 37°C (pH 7.4) [12], [13], [14]. Hearts were retrogradely perfused at a constant pressure and ventricular pressure was monitored with a fluid-filled balloon placed in the left ventricle (LV) and connected to a 1.4Fr Millar pressure catheter (model SPR-671, Millar Instruments, Houston, TX). Volume of the balloon was adjusted to 10 mmHg and hearts were electrically paced via the right atrium at a rate of approximately 450 beats/min. During each experiment, the heart was allowed to stabilize for 20 minutes and baseline values were collected at the end of stabilization period. Hearts were then exposed to 30 min of global ischemia followed by 60 min of reperfusion. During ischemia, temperature was carefully kept at 37°C. Pacing was stopped 1 min prior to start of ischemia and restarted 5 min after the start of reperfusion. Developed pressure was recorded throughout the experimental protocol.

### Ischemia/reperfusion and infarct measurements

Male mice approximately 4–9 months were used for *in vivo* ischemia/reperfusion experiments (5 control and 7 αMHC-tTA) according to the American Physiological Society Guiding Principles for the Care and Use of Animals in Research and Training. Mice were initially anesthetized with isoflurane (4%) followed by administration of etomidate (10 mg/kg BW; i.p.). Subsequently, mice were intubated with an 18 gauge angiocath sleeve and surgical anesthesia was maintained using 1.5% isoflurane delivered through a vaporizer with 100% oxygen (compressed gas). The vaporizer was connected in series to a rodent ventilator (Harvard Instruments) with the stroke volume set at 0.2 to 0.3 ml/min (based on body weight) and a respiration rate of 135 per minute. A left thoracotomy was performed to expose the heart and the pericardium was ruptured to access the left anterior descending (LAD) coronary artery. The LAD was occluded with 8-0 monofilament nylon suture approximately 5 mm from the ostium and the thoracotomy was temporarily closed and covered with saline-soaked gauze while the animal remained under anesthesia and ventilated. After 45 minutes of occlusion, the suture was removed and the LAD coronary bed was reperfused for an additional 120 minutes under anesthesia and ventilation. At the conclusion of the reperfusion period, the mouse was euthanized by overdose with isoflurane anesthesia and the heart was explanted. To identify normal myocardium, the heart was perfused retrograde through an aortic cannula with ice-cold PBS and the LAD was re-occluded under a dissecting microscope at the same site as performed for the *in vivo* experiments. Evans Blue dye (1% in PBS) was then perfused retrograde through the coronary vasculature and stained the entire heart except for the LAD coronary bed. The hearts were then detached from the cannula, placed in an acrylic heart matrix (Aster Industries, McCandles, PA) and cooled at −20°C for an additional 30 minutes. To identify the area at risk (AAR) and infarcted area (IA), the heart was cut from apex to base into seven, 1 mm thick slices and each slice was incubated at 37°C for 25 min with 2% 2,3,5-triphenyltetrazolium chloride (Sigma Aldrich) dissolved in PBS. Slices were fixed overnight in 10% buffered formalin for analysis. To determine the AAR and IA, each slice was scanned and digitized. AAR and IR were calculated for each of seven slices per heart in the control and αMHC-tTA groups by planimetry as previously reported [15], [16] and expressed as a percentage of the entire cross-sectional area (Evans Blue+AAR+IA) for each slice.

### Creatine kinase release

Creatine kinase activity was determined in perfusion buffer collected from the heart at baseline, at the onset of ischemia, and at 15, 30, 45 and 60 min of reperfusion. Enzyme activity was measured using a colorimetric assay using EnzyChrom Creatine Kinase Assay Kit (ECPK-100; BioAssay Systems, CA).

### Statistical analysis

All results are presented as mean ± SEM. Statistical significance was determined by a one-tailed Student's t-test assuming unequal variance. A p value<0.05 was considered significant.

## Results

### Echocardiography and *in vivo* cardiac function

α-MHC-tTA and control mice were analyzed by echocardiography to determine LV wall thickness, fractional shortening and ejection fraction. Table 1 shows that LV end-diastolic dimension and end-diastolic posterior and septal wall thickness were not changed in α-MHC-tTA compared to control. HR was similar in both α-MHC-tTA and control. Furthermore, a slight increase in fractional shortening and ejection fraction was observed in α-MHC-tTA animals compared to control but these differences were not statistically significant. There were no notable changes in either LV mass or in HW/TL ratio between the two groups indicating lack of hypertrophy in the α-MHC-tTA animals compared to control. To further test whether hypertrophy developed over time, two older α-MHC-tTA mice (12 and 14 months, respectively) were examined by echocardiography and found to lack evidence of hypertrophy (data not shown). This 14 month old animal was initially examined at 7 months (included in Table 1) and found to lack signs of hypertrophy. Thus, our results indicate an absence of compensatory hypertrophy in the α-MHC-tTA mice.

**Table 1:** Echocardiographic Measurements and Heart weight/tibia length.

	tet	MHC-tTA	P value
N	6	5	
**LV end-diastolic dimension, mm**	3.9±0.2	3.7±0.2	0.38
**LV end-systolic dimension, mm**	2.7±0.2	2.3±0.1	0.16
**End-diastolic septal wall thickness, mm**	1.0±0.06	1.0±0.05	0.26
**End-diastolic posterior wall thickness, mm**	0.9±0.03	0.9±0.05	0.38
**Heart rate, beats/min**	483±33	510±12	0.19
**Fractional shortening, %**	32±3.0	39±1.2	0.06
**Ejection fraction, %**	63±4.0	71±1.7	0.13
**LV mass, mg**	114±9	115±7	0.46
**HW/TL (mg/mm)**	7.9±0.4 (n = 5)	8.3±0.3 (n = 12)	0.23

Values are mean ± SEM. *P* values are based on 1-tail Student's *t*-test assuming unequal variance.

### Ischemia-reperfusion injury in the isolated perfused heart

**Figure 1:**
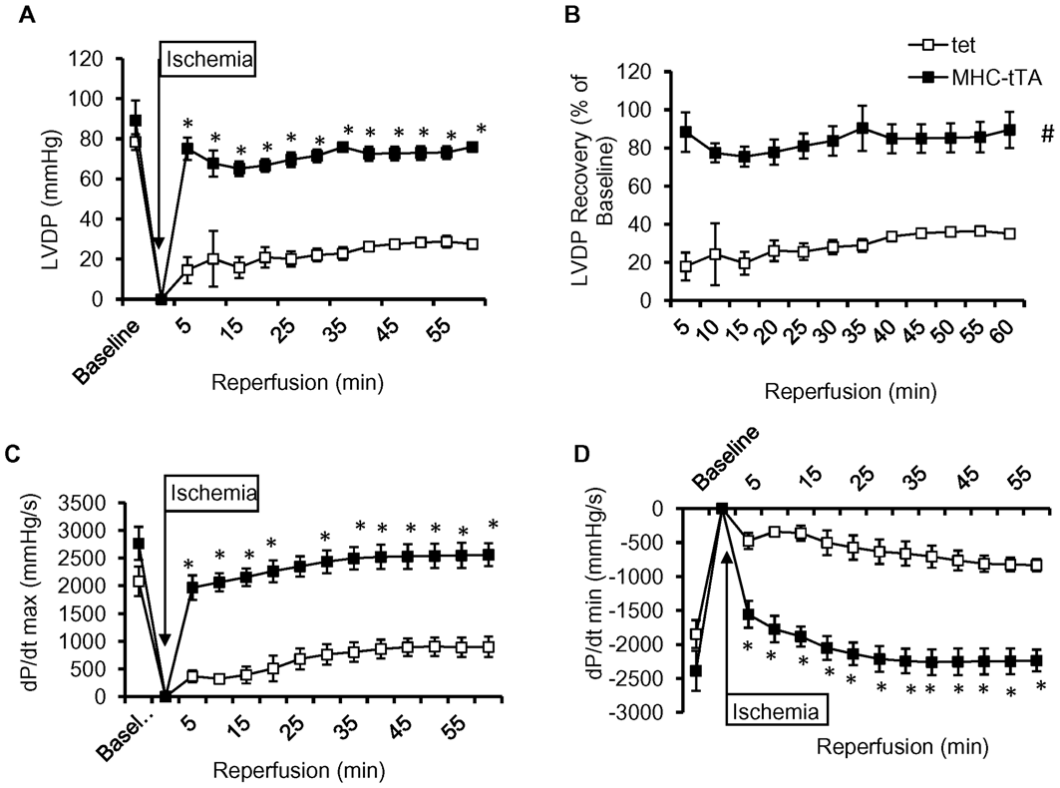
α-MHC-tTA hearts were protected against I/R injury *in vitro* using the Langendorff-perfused heart. (A–B) LVDP was similar between α-MHC-tTA and control hearts at baseline; however, after 30 min of ischemia, control hearts recovered to 35% of baseline LVDP values whereas α-MHC-tTA had 90% recovery. (C–D) LV systolic and diastolic functions assessed by dP/dt_max_ and dP/dt_min_ respectively were significantly higher in α-MHC-tTA compared to control after 30 min of ischemia. (N = 3 for control and N = 4 for αMHC-tTA, p<0.05).

No-flow ischemia was performed for a total of 30 min in α-MHC-tTA and control hearts followed by 60 min reperfusion. Figure 1A shows LVDP at baseline, during ischemia, and from 5 to 60 min reperfusion. The control group showed a maximum recovery of 35% whereas α-MHC-tTA had a robust recovery of 90% compared to baseline (Figure 1B). The time to return to 90% of baseline LVDP was almost immediate (within the first 5 min of reperfusion). Furthermore, LV systolic and diastolic functions assessed by dP/dt_max_ and dP/dt_min_, respectively, were significantly higher in α-MHC-tTA compared to control after ischemia (Figure 1C and D). When both groups were subjected to 40 min ischemia, recovery in the α-MHC-tTA was at 55% of baseline versus 12% in control (data not shown). These results demonstrate a sustained preconditioning effect in α-MHC-tTA that is comparable to ischemic preconditioning seen with brief periods of ischemia followed by reperfusion.

### PI3K inhibitor in ischemia-reperfusion injury

**Figure 2:**
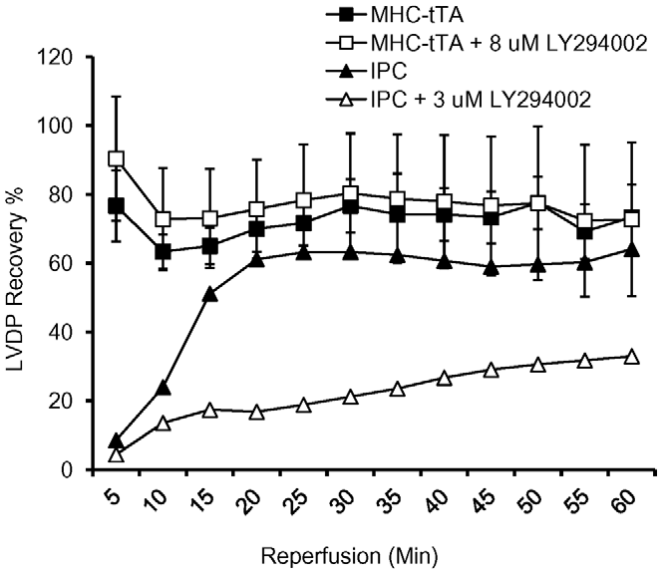
Effect of PI3K inhibition on the protection against I/R injur in α-MHC-tTA hearts. Administration of LY294002 did not abolish the protective effect seen in α-MHC-tTA hearts however it did abolish protection imparted by IPC. (N = 5 for α-MHC-tTA and N = 3 for α-MHC-tTA+8 µM LY294002, p<0.05).

Administration of LY294002, a selective PI3K inhibitor, at 3 µM was sufficient to decrease the preconditioning effect induced by two cycles of 5 min ischemia/5 min reperfusion prior to 30 min ischemia in control hearts (Figure 2). However, treatment with 3 µM (data not shown) or 8 µM LY294002 did not abolish preconditioning in the α-MHC-tTA hearts.

### Creatine phosphokinase release as a measure of cardiomyocyte damage

**Figure 3:**
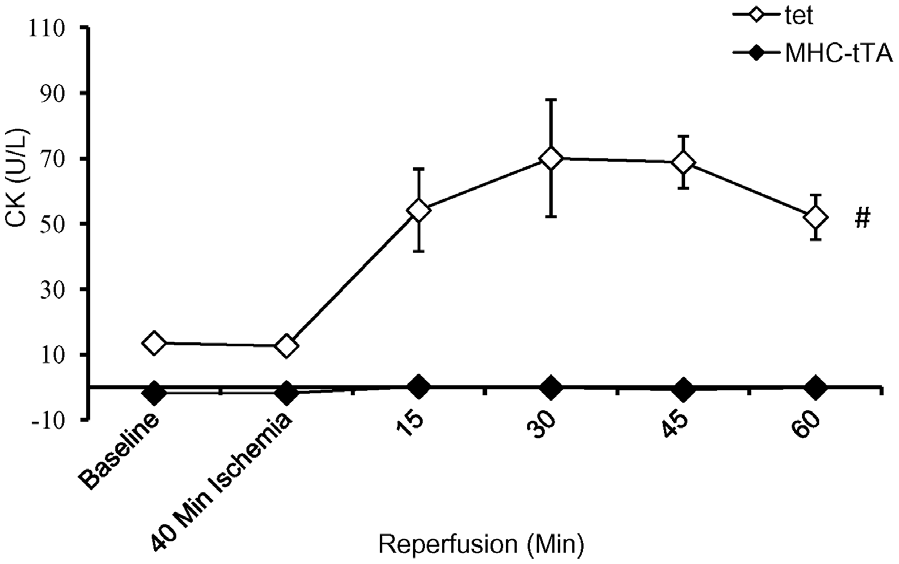
Reduced cardiac muscle damage in α-MHC-tTA hearts subjected to I/R injury. Lack of cardiac muscle cell damage was apparent in α-MHC-tTA compared to control where abundant creatine kinase levels were observed within 15 min of start of reperfusion. (N = 4, p<0.05).

The release of creatine phosphokinase (CK) into perfusion buffer is a marker for cardiomyocyte damage [17]. As shown in Figure 3, control hearts exhibit significant elevated CK in perfusion buffer within 15 min of reperfusion following ischemic injury. In contrast, CK activity in perfusion buffer from α-MHC-tTA hearts was at or below baseline levels. The difference in baseline CK levels is possibly due to cardiac muscle damage during heart preparation for the experiment further highlighting the increased resilience of the α-MHC-tTA hearts.

### Coronary artery ligation

**Figure 4:**
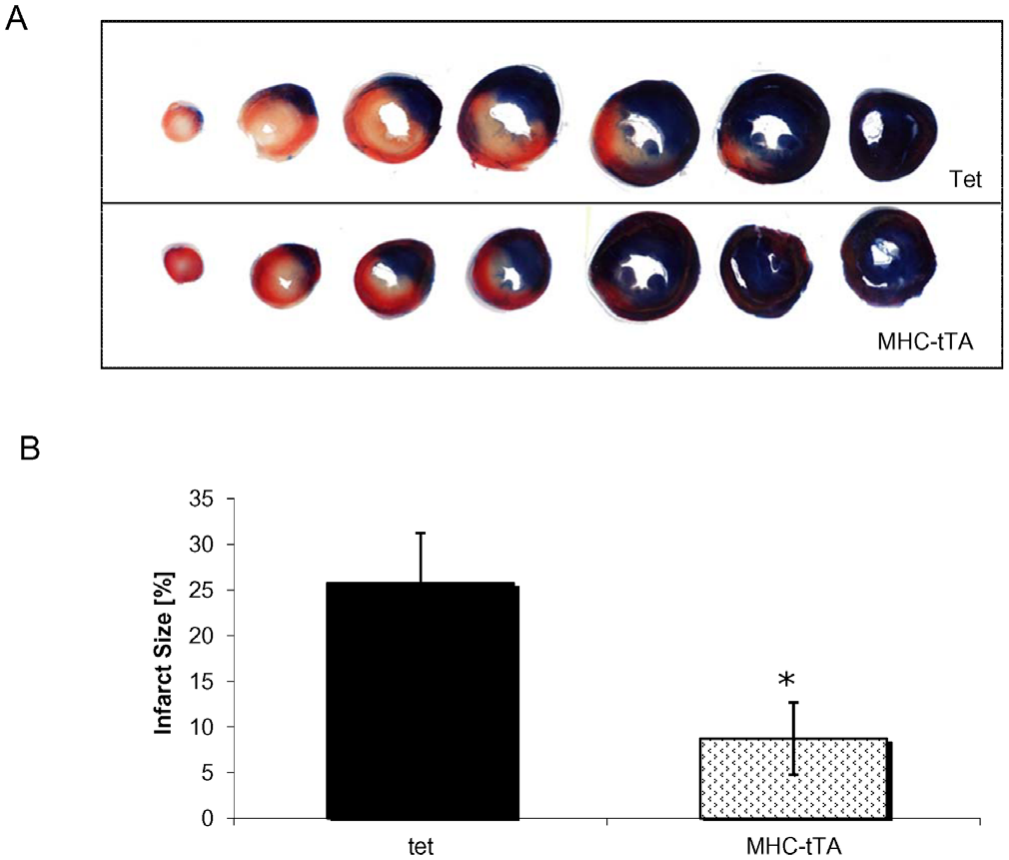
Effect of *in vivo* I/R injury on α-MHC-tTA hearts. Significantly smaller infarct sizes were observed in α-MHC-tTA hearts subjected to 45 min of left coronary artery occlusion followed by 120 min of reperfusion. (A) Representative cross sections from control and α-MHC-tTA hearts. (B) Average infarct size in control and α-MHC-tTA hearts. (N = 5 for control and N = 7 for α-MHC-tTA, p<0.05).

To assess whether the cardioprotection observed in the α-MHC-tTA mice extended into an *in vivo* setting, we subjected animals to a coronary artery ligation model. Control and α-MHC-tTA animals were subjected to 45 min of left coronary artery occlusion followed by 120 min of reperfusion. As shown in Fig. 4, the size of infarct compared to the area-at-risk (AAR) was reduced by 66% in the α-MHC-tTA animals compared to control mice. Thus, the α-MHC-tTA mice exhibit significant protection from cell death *in vivo*.

## Discussion

The rat α-MHC-tTA mouse model is widely used to assess the role of various gene products on cardiac function. However, previous studies reported that this model exhibits significant cardioprotection from I/R injury along with an associated cardiac hypertrophy and mild cardiomyopathy [8], [9]. In our attempts to use this model to assess the function of the intersectin scaffold protein in cardiac biology, we observed that the α-MHC-tTA mice exhibited significant cardioprotection. In contrast to previous studies [8], [9], however, our mice lacked the reported cardiac hypertrophy and cardiomyopathy phenotypes. Hemodynamic measurements of control and α-MHC-tTA mice revealed no significant differences in the baseline cardiac properties of these animals. Furthermore, heart weight to tibia ratios as well as echocardiographic measurements indicated that the α-MHC-tTA mice lacked an associated hypertrophy phenotype.

Another major finding of our study is that we demonstrate for the first time that the α-MHC-tTA mice exhibit significant cardioprotection from cell death *in vivo*. We observed a 66% reduction in infarct size in α-MHC-tTA mice compared to control animals following occlusion of the coronary artery and reperfusion. Thus, α-MHC-tTA mice represent a potentially useful model for discovery of novel factors involved in cardioprotection. Given the differences in genetic background of our animals (C57Bl/6) versus those of Baker and colleagues (FVB/N), it is possible that there are genetic modifiers to this phenotype (i.e., hypertrophy and cardiomyopathy) and that further characterization of the α-MHC-tTA mice on different genetic backgrounds may lead to the identification of such modifiers.

Cardioprotection for I/R injury can be induced by ischemic preconditioning (IPC) which involves brief intermittent periods of ischemia and reperfusion. There are two phases of IPC protection: the acute phase which lasts 2 hrs and a delayed phase which occurs 24 hrs post-ischemic injury [18]. The activation of PI3Ks is thought to mediate the acute and the delayed phases of IPC by phosphorylation of Akt, PKC (involved in acute phase) and PDK1 (involved in delayed phase). The cardioprotective phenotype of the α-MHC-tTA hearts is qualitatively different from that induced by IPC. In the latter paradigm, the heart gradually increases its contractility upon start of reperfusion and reaches a maximum plateau that is approximately 60% of baseline. In contrast, the α-MHC-tTA hearts reach 70–80% function within 5 min of reperfusion. In addition, the phenomenon of postconditioning is thought to occur via Akt activation [19]. PI3K inhibitors (wortmannin and LY294002) abolish the protection of both acute and delayed IPC [18]. However, we report here for the first time the lack of sensitivity of α-MHC-tTA cardioprotective phenotype to PI3K inhibition. In addition, we did not observe changes in the phosphorylation status of Akt in α-MHC-tTA hearts (data not shown) further supporting the conclusion that the PI3K pathway is not involved in α-MHC-tTA-stimulated cardioprotection. There is a possibility that the PI3K/Akt pathway was activated early in development in which case an increase in organ size should have been observed during adulthood [20]. However, there were no notable changes in heart size observed in the α-MHC-tTA.

Earlier investigations into the molecular pathways involved in α-MHC-tTA phenotype revealed alterations in the expression of 153 genes [8]. Among these genes are myofilament proteins such as β-tropomyosin and skeletal α-actin as well as genes involved in intracellular transport, intracellular signaling in addition to kinase, transferase and heat shock protein activities. Inhibition of tTA function with doxycycline did not change the gene expression profile. Furthermore, the cardiac tet-inducible system has been recently reengineered using the attenuated mouse α-MHC promoter to drive low level expression of tTA [21]. This modified system induces robust expression of cardiac contractile and signaling proteins in the absence of an overt phenotype such as the one observed in this as well as other studies. One possible explanation for these observed differences between the two MHC-tTA promoters is that insertion of the rat α-MHC-tTA transgene into the genome altered genetic elements (e.g., genes or micro RNAs) that contribute to cardioprotection. Thus, identification of the transgene insertion site may shed insight into the molecular mechanisms involved in the observed gain-of-function phenotype observed in the current study.

Although the tet-regulated system provides an attractive model for chemical regulation of transgene expression, caution must be used when utilizing the rat α-MHC-tTA transgenic model in the study of cardioprotection due to the significant cardioprotective response observed in the α-MHC-tTA alone. We strongly encourage investigators to include these single transgenic animals as controls for comparison to double transgenic animals. However, our studies reveal that the cardioprotective phenotype associated with the α-MHC-tTA transgene is not associated with any adverse cardiac remodeling or dysfunction. Thus, identification of the molecular mechanism underlying this cardioprotective phenotype may uncover novel molecular targets for therapeutic intervention during ischemic injury.
